# Postmortem Study of Molecular and Histological Changes in the CA1 Hippocampal Region of Chronic Methamphetamine User

**DOI:** 10.22037/ijpr.2019.15483.13123

**Published:** 2019

**Authors:** Gholam-Reza Mahmoudiasl, Hojjat Allah Abbaszadeh, Mostafa Rezaei-Tavirani, Mohammad-Amin Abdollahifar, Yousef Sadeghi, Maryam Sadat Khoramgah, Somayeh Niknazar, Shahram Darabi

**Affiliations:** a *Hearing Disorders Research Center, Loghman Hakim Hospital, Shahid Beheshti University of Medical Sciences, Tehran, Iran. *; b *Laser Application in Medical Sciences Research Center, Shahid Beheshti University of Medical Sciences, Tehran, Iran. *; c *Proteomics Research Center, Faculty of Paramedicine, Shahid Beheshti University of Medical Sciences, Tehran, Iran. *; d *Department of Biotechnology, School of Advanced Technologies in Medicine, Shahid Beheshti University of Medical Sciences, Tehran, Iran. *; e *Cellular and Molecular Research Center, Faculty of Medicine, Qazvin University of Medical Sciences, Qazvin, Iran.*

**Keywords:** Methamphetamine, CA1, Hippocampous, GFAP, Iba1, Apoptosis

## Abstract

Methamphetamine (Meth) is recognized as one of the most important new distributed abused drug that causes severe damage to the different parts of the brain, especially hippocampus. Previous studies have demonstrated that Meth can induce apoptosis and cell death in the brain. In this study, we evaluated the long-term effects of Meth abuse in the CA1 region of postmortem hippocampus. Postmortem molecular and histological analysis was performed for five non-addicted subjects and five Meth addicted ones. Iba-1 (microglia) and glial fibrillary acidic protein, GFAP (astrocytes) expression were assayed by western blotting and immunohistochemistry (IHC) methods. Histopathological assessment was done with stereological counts of hippocampal cells stained with hematoxylin and eosin (H and E). Tunel staining was used to detect DNA damage in human brains. In addition, protein-protein interaction analysis network was investigated. Western blotting and immunohistochemistry assay showed overexpression of GFAP and Iba-1 protein in the CA1 hippocampal region of Meth users’ brain. Stereological analysis in the CA1 region revealed increased neuron degeneration. Furthermore, significant apoptosis and cell death were confirmed by Tunel assay in the hippocampus. The prominent role of TLR4, IL1B, CASP1, and NLRP3 in the molecular mechanism of Meth was highlighted via PPI network analysis. Chronic Meth use can induce GFAP and Iba-1 upregulation and neuronal apoptosis in the CA1 region of the postmortem hippocampus.

## Introduction

Methamphetamine (Meth) is a new recreational drug. Based on the USA Food and Drug Administration, approximately 38 million people are reported as having an addiction to Meth and/or its related derivatives. Unexpectedly, Meth is the most commonly used substance after Cannabis (1). It is known as the sympathomimetic drug that has been reported to cause several physical and psychological side effects such as un Normal able repetitive movements, sweating, pupil dilation, and severe behavioral reactions (2). It also causes behavioral consequences including sensitization, Meth discriminative stimulus effects, and hyper motor activity by inducing neuroinflammation. Based on several studies, behavioral effects may be caused by the role of Meth in regulating the level of 3′-5′-cyclic adenosine monophosphate (cAMP). It could be responsible for the behavioral effects of Meth such as hyper motor activity. Meth has also a role in mediating inhibition of phosphodiesterase (PDE), the enzyme that is responsible for cAMP degradation (3). Meth severely damages different regions of the brain (4). Its chronic use may lead to neurodegeneration of cortex, hippocampus and midbrain areas (5). Severe symptoms of Meth abuse can be due to hippocampal-dependent memory changes (6). Several experimental studies have demonstrated that astrocytes and microglia are stimulated in rodents which were treated by a toxic Meth regime (7-9). Meth exposure is associated with microglial activation and along with that, it induces secretion of proinflammatory cytokines and ultimately causes drug induced-behavioral changes which could be attenuated by modulation of activated glial cells (10). Previous studies have revealed astrocyte activation in Meth-induced toxicity (11, 12). Some researchers showed Meth-induced toxicity was related to dramatic elevation in the levels of GFAP which was more prominent in the striatum and interestingly this sub-region is more vulnerable to the toxic effects of Meth. The loss of dopamine-transporter binding sites and the immune reactivity of tyrosine-hydroxylase are the most in the striatum (13). Based on astrogliosis analysis among Meth-treated animals, it is evident that the astroglial response reaches its peak within 2 days after administration and remains high for at least 7 days (14). Furthermore, a correlation was observed between the Meth-induced activation of astrocytes and toxicity (15). In fact, after Meth treatment, astrocyte can actively respond in a short period of time and this response can be relatively prolonged. Since astrocytes have phenotypic changes capability and dynamic response potential, they can play an important role in the neuropathological consequences of CNS injuries (16). The Meth-induced microglial responses, for instance, expresses calcium binding adaptor protein (Iba-1) that may be responsible for the neuropathological alterations secondary to neurotoxic effects of Meth (17). Extensive research evidence indicates that attenuation of microglial activation can decrease Meth- induced behavioral changes (18-20). For instance, Ibudilast (3-isobutyryl-2-isopropylpyrazolo [1,5- a] pyridine) is a non-selective PDE inhibitor and anti-inflammatory glial cell modulator that can attenuate Meth-induced locomotor activity and its sensitization in mice (21). As well, treatment with minocycline hydrochloride (other anti-inflammatory drugs) significantly reduces microglial activation caused by Meth and attenuates Meth-induced behavioral deficits (22, 23). The purpose of this study is to explore whether chronic Meth use can induce GFAP and Iba-1 upregulation and neuronal apoptosis in the CA1 region of the postmortem hippocampus.

## Experimental

The brains of meth user and non-meth user cadavers were obtained from the Iranian Medical Jurisprudence organization for post mortem examination (Tehran, Iran). We collected and preserved the human brains in a way that is in accordance with the Declaration of Helsinki. All protocols were approved by the Ethics Committee of Shahid Beheshti University of Medical Sciences (IR.SBMU.RETECH.REC.1396.542). The analysis was done on 14 chronic male Meth users (aged 39 ± 1.9 years) who died of a drug overdose with the average consumption duration of more than 5 years (The duration could affect the amount of damage of the hippocampus). The Normal groups consisted of 12 male adults without a history of Meth use (aged 38 ± 2 years). Normal and Meth subjects matched in terms of age and post mortem delay (duration of autolysis). None of the Meth users had a history of mental illness or neurodegenerative disorders. Moreover, none of them was HIV positive. Body mass index analysis (kg/m^2^) was done for all of the samples. 

**Figure 1:**
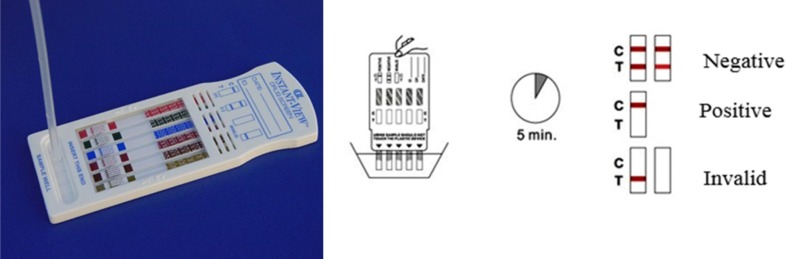
Kit detection for Meth in the human urine sample. The band is prepared for detecting Meth in human urine. This assessment is a type of qualitative test and thus, it delivers a ″Negative″ or ″Positive″ consequence

The urine Kit detection for Meth was used for the samples (Identify Diagnostics 5). The Meth urine test will be positive only if the urine sample contains Meth (Figure 1).

*Sampling and tissue preparation*

**Figure 2:**
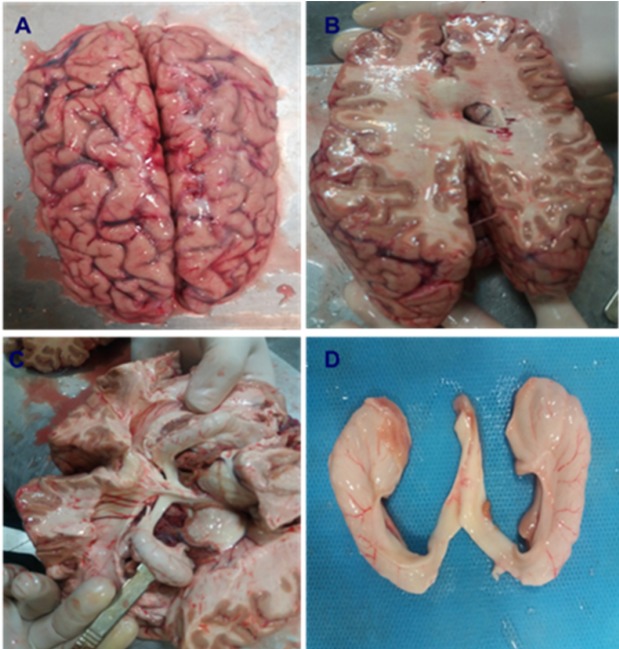
Brain dissection and isolation of hippocampus in Normal and Meth groups

After obtaining the brain donation consent from the families of the donors, freshly isolated brain tissues of 14 documented Meth users and 11 Normal cadavers were transported to the lab kept in Ringer Lactate solution. The samples were weighed just after removing blood clots and necrotic tissue. Then, the CA1 region of the hippocampus was exposed and removed from the brain (Figure 2). Afterward, the tissue samples were fixed in 4% Paraformaldehyde for 1 week in order to prepare the samples for Hematoxylin and Eosin staining (H and E), Tunel assay and immunohistochemistry. The CA1 region was placed into paraffin blocks and microtome was used to cut it in its longitudinal axis into several 5 and 25 μm thick sections. The slides were stained using H&E, Nissl and Golgi techniques.

*Semi Quantitation of GFAP and Iba1 protein expression analysis (Western blotting)*

Proteins expressions of GFAP, Iba-1, and GAPDH in the CA1 region of the hippocampus were analyzed by using western blotting assay. The tissues were washed two times with PBS at first and then were squished and mixed with buffer and centrifuged for 15 min. The proteins were separated by electrophoresis using sodium dodecyl sulfate-polyacrylamide gel (SDS-PAGE). Afterward, they were moved to polyvinylidene difluoride (PVDF) and finally, they were examined with primary antibodies including rabbit polyclonal anti-GFAP (1:200), and human polyclonal anti-Iba1 (1:200). The secondary antibodies conjugated with horseradish peroxidase (HRP) (Cell Signaling Technology, USA). For internal Normalling, the GAPDH generation signal was used (24). Finally, Western Blot data were quantified by using ImageJ Software.

*Immunohistochemistry*

The brains were post-fixed (1 Week) with 4% paraformaldehyde and then transferred to 30% sucrose (Sigma-Aldrich, Germany) solution until reaching equilibration. Afterward, the hippocampus segments were sectioned (8 μm in thickness) and stored at -20 °C in a cryoprotectant buffer containing 25% ethylene, 25% glycerin, and 0.05 M phosphate buffer (all from Sigma-Aldrich, Germany). The slices were incubated by human monoclonal anti-GFAP and anti-Iba1 (Abcam, USA) antibodies and diluted to 1:100 overnight in the primary reaction. Subsequently, this process was followed by a similar washing with PBS, and 1-hour incubation with goat anti-mouse FITC-conjugated secondary antibody (ab6785, Abcam, USA) at a 1:100 dilution in the second reaction. The tissue sections were washed with PBS, and the nuclei were counterstained by using DAPI (25).

*Stereological study*

*The length of dendrite Estimation*

To estimate the length, a vertical section was considered. A grid was superimposed on the microscopic images of the hippocampus. Using a microscope (Nikon E-200) connected to a computer, the dendrite lengths were measured by the following Equation:

$l_{N}=2.\frac{a}{l}.\frac{a}{\mathrm{asf}}.M^{-1}.\frac{\sum I}{\sum Q}$

where Q is the cell bodies of the neurons, I is the total number of intersections, and M is magnification (26).

*Estimating the volume of the hippocampus*

Using the stereological software, a grid of points was superimposed on the images. The Cavalieri method was used as an estimator of the hippocampus volume. The volume of the hippocampus was measured by the following Equation:

$V_{\mathrm{total}}=\sum P\times t\times \frac{a}{p}$

Where ƩP is the total point hitting the hippocampus. a/p is the area associated with point and t is the distance between the sample section (26).

*Neurons and glial cells number Estimation*

A counting frame grid was superimposed on the microscopic images. Then, a microcator was attached to the stage of the microscope to measure the z-axis in the tissue. The number of neurons and glial cells was determined by the optical dissector technique using the subsequent Equation (27):

$\mathrm{Nv}=\frac{\sum Q}{\sum P\times \frac{\alpha }{F}\times h}\times \frac{t}{\mathrm{BA}}$

In this Equation, ΣQ-” is the number of cell counted, “ΣP” is the total number of the microscopic fields, a/f is the area per frame, “h” is the height of the director, “t” is the real section thickness, and BA was the block advance of the microtome. 

The total number of the neurons was estimated by multiplying the numerical density (Nv) by the V (total).

*Tunel assay*

Tunel assay was performed by using *in-situ* Cell Death Detection kit (fluorescence, Roche, CH). The CA1 region of the hippocampus was fixed, placed within paraffin, and mounted on glass slides. 

Then, the paraffin was removed by using Xylene and then rehydrated in a piecemeal series of ethanol. After washing it with water, the Tunel staining was performed and the Tunel protocols were applied. The results of Meth and Normal groups were evaluated by using ImageJ software.

*Protein network*

The genes were separately and both together accompanied by 50 relevant genes proceeded via protein query of STRING database. The PPI network was constructed by Cystoscope software version 3.6.0. Confidence = 0.4 and the undirected links were considered.

*Statistical analysis*

The statistical analysis was done using IBM SPSS software. The significance level was analyzed using the analysis of variances (ANOVA). 

Moreover, for another statistical comparison of multiple means in the groups, One-way ANOVA and Tukey’s post hoc test was performed. 

## Results

*Body mass index decreased in Meth users*

**Figure 3.**
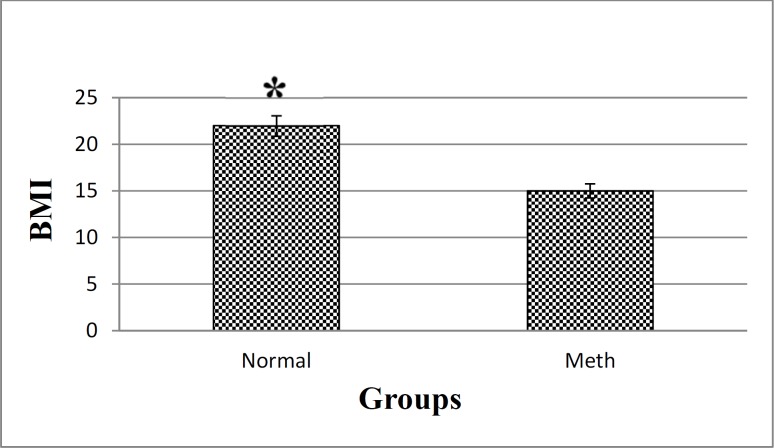
BMI in Meth and Normal groups. The results showed that a significant decrease in the BMI of Meth groups compare to Normal groups.*P < 0.05

The body mass index (BMI) is an indicator of weight status, and it is calculated by dividing weight in kilograms relative to the square of height in meter. There could be a potential association between Meth exposure and BMI. In our study, the BMI analysis demonstrated that there is a change in the BMI of Meth users and normal groups (Figure 3). Based on the results, a decrease in the BMI index is evident in the Meth groups comparing to the Normal groups (*P *< 0.05).

*GFAP and Iba-1 protein levels are elevated in the CA1 hippocampal region in Meth users*

**Figure 4.**
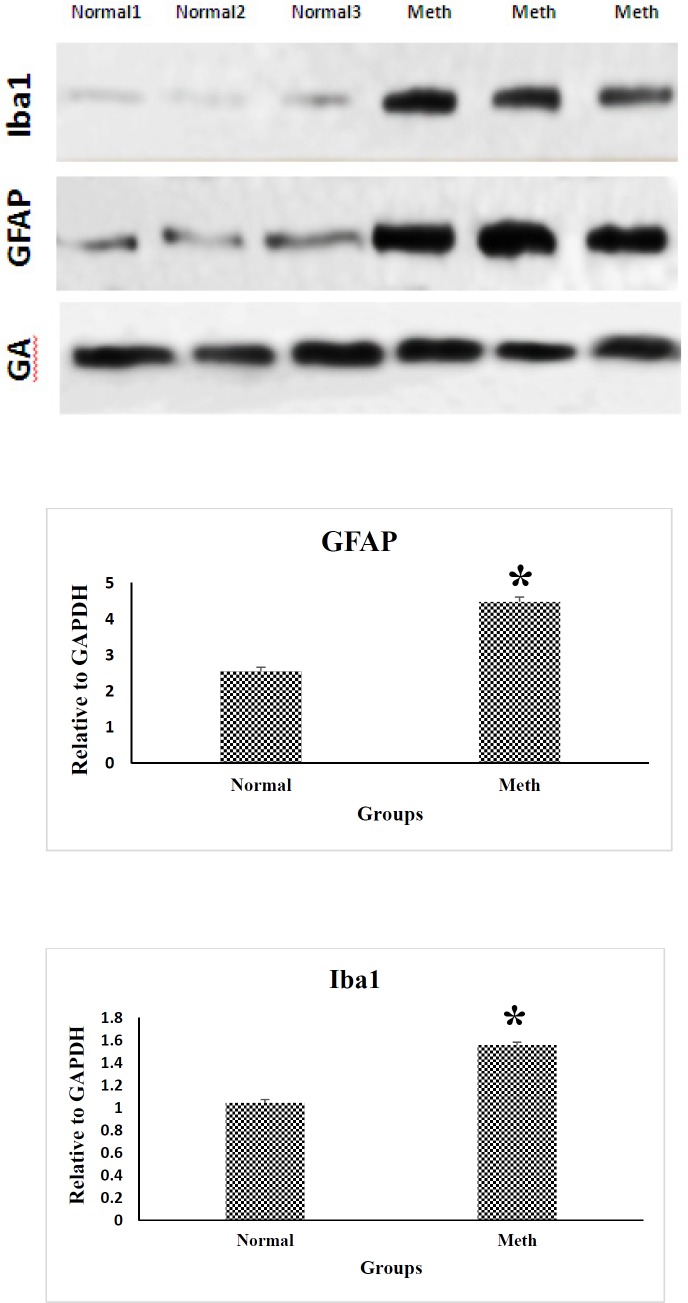
Western blotting analysis of GFAP and Iba1 proteins in the CA1 region of the hippocampus in Meth and Normal groups. GFAP and Iba-1 protein levels are increased in the CA1 hippocampal region in Meth groups comparing to the Normal groups (^*^P < 0.05)

The protein expression of Iba-1 and GFAP in the CA1 region of the hippocampal brain samples are shown in Figure 4 in the normal and Meth groups. Iba-1 and GFAP expression level increased by 2-fold in the CA1 hippocampal region of the Meth addicted brains in comparison with the Normal groups. Western Blot data is quantified by using ImageJ Software (*P* < 0.05).

*Immunohistochemical of GFAP and Iba-1 increased in the hippocampal region in Meth users*

**Figure 5:**
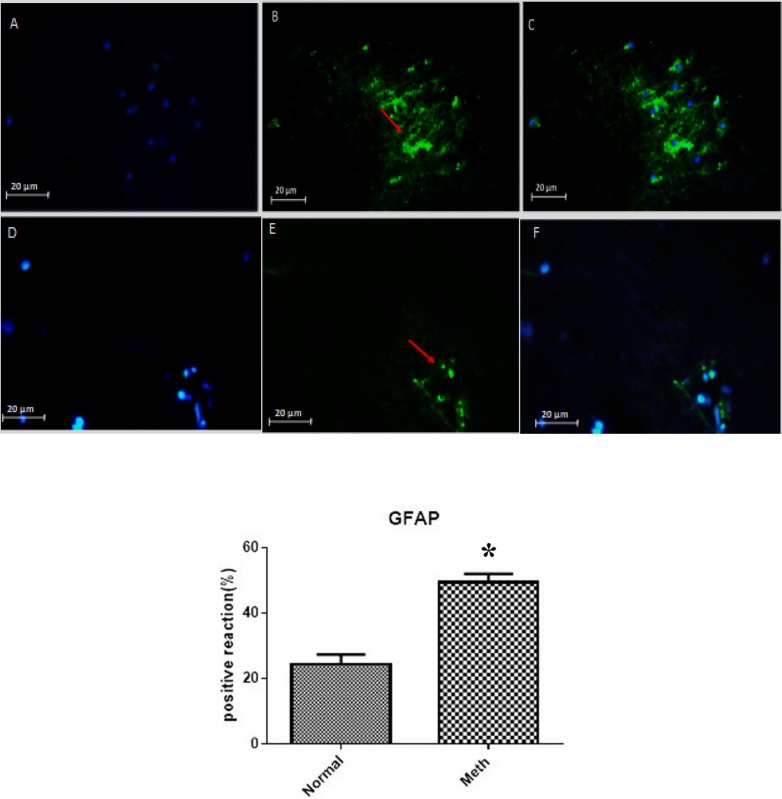
The expression of GFAP in Meth and Normal groups are shown in the upper row (Meth groups) and lower row (Normal groups). (A and D) nuclei stained by DAPI (Blue). (B and E) primary antibody to GFAP (Green). (C and F) merge. GFAP protein level increased in the CA1 region of the hippocampus in the Meth groups compared to Normal groups (^*^P < 0.05).

**Figure 6:**
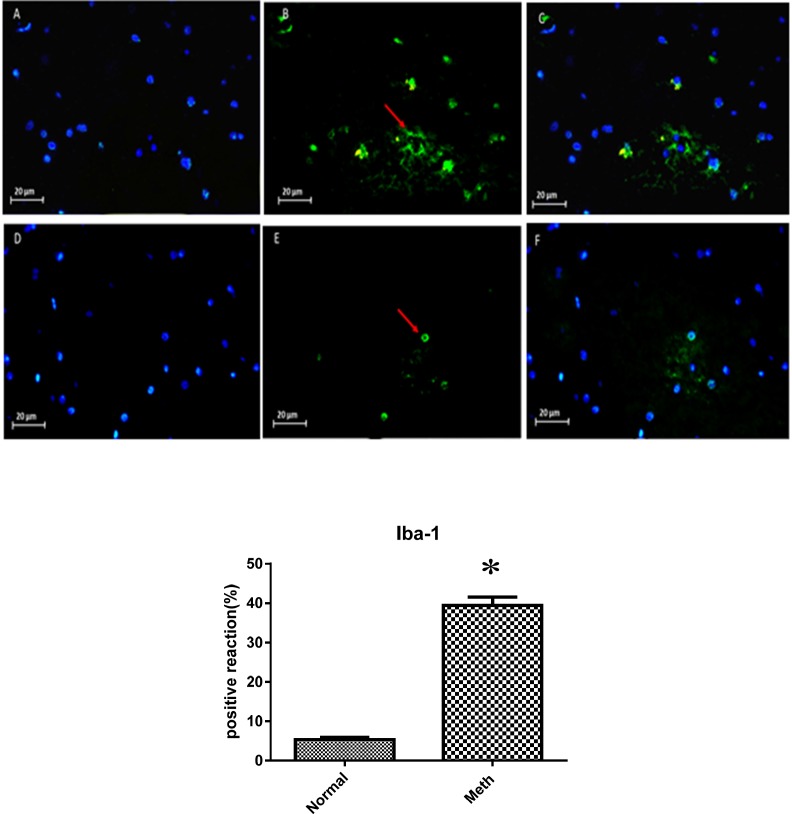
Expression of Iba-1 in Meth and Normal groups, upper row (Meth groups) lower row (Normal groups) (A and D) nuclei stained by DAPI (Blue). (B and E) primary antibody to Iba-1(Green), (C and F) merge. Iba-1protein levels increased in the Meth groups compared to Normal groups (^*^P < 0.05)

The CA1 region of the hippocampus in both Meth and Normal groups was investigated by using anti- GFAP and Iba-1 antibodies in order to detect the presence of these proteins in the re-filled areas (Figures 5 and 6). 

As shown in the figures, the expression of these protein markers was elevated in the CA1 hippocampal region of the Meth groups compared to the Normal groups (*P *< 0.05).

**Figure 7:**
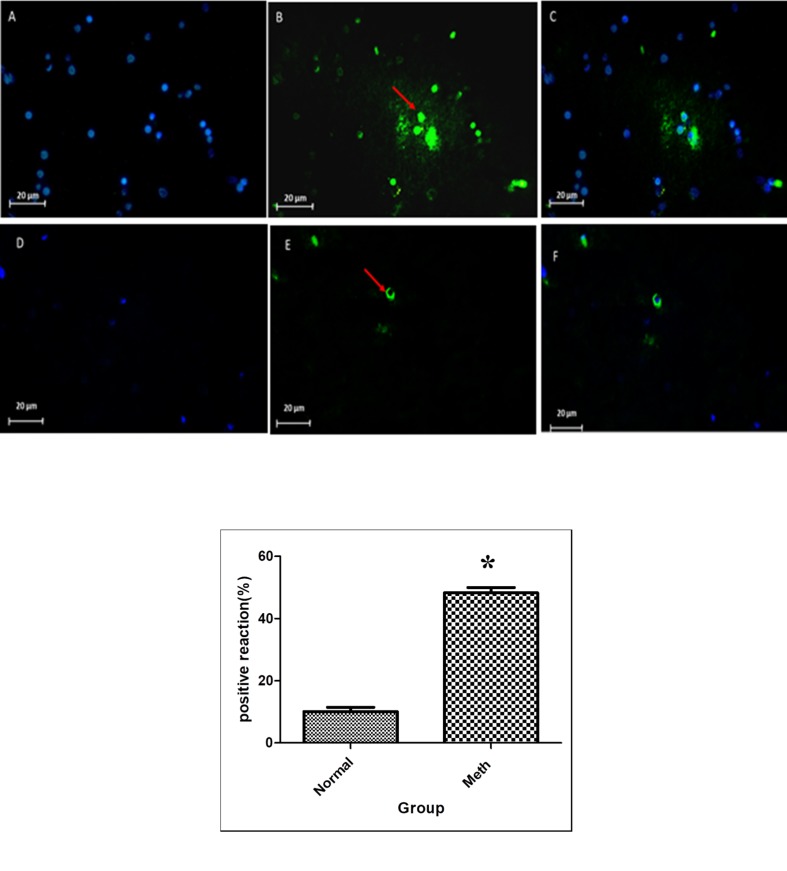
Tunel Assay in Meth and Normal groups, upper row (Meth groups) lower row (Normal groups). *P *< 0.05. The apoptosis increased in the CA1 region of the hippocampus in the Meth groups compared to the Normal. (A and D) nuclei stained by DAPI (Blue). (B and E) apoptotic cells (Green). (C and F) merge. The arrow shows the apoptotic cells

The nuclei were stained using DAPI. The apoptotic cells were Tunel positive and were merged with positive reaction cells (Figure 7). The results of the Tunel test demonstrated that the average of Tunel positive cells count (10^3^ mm^2^) was 50 % in the Meth groups and 15 % in the Normal groups.

*The volume of hippocumos and the number of neuron reduced and the number of the glial cells increased in the hippocampus in Meth users*

The number estimation (stereological analysis) was done by H and E staining. The results indicated that the density of the cells is significantly different between the Meth and Normal groups. The number of the cells in the Meth groups was significantly less than that in the Normal groups. 

The total volume of the hippocampus was significantly decreased in the Meth groups in comparison with the Normal groups (*P* < 0.001). The total number of neurons in Meth groups decreased and the total number of glial cells increase in Meth groups in comparison with the Normal groups (*P* < 0.001). 

**Figure 8:**
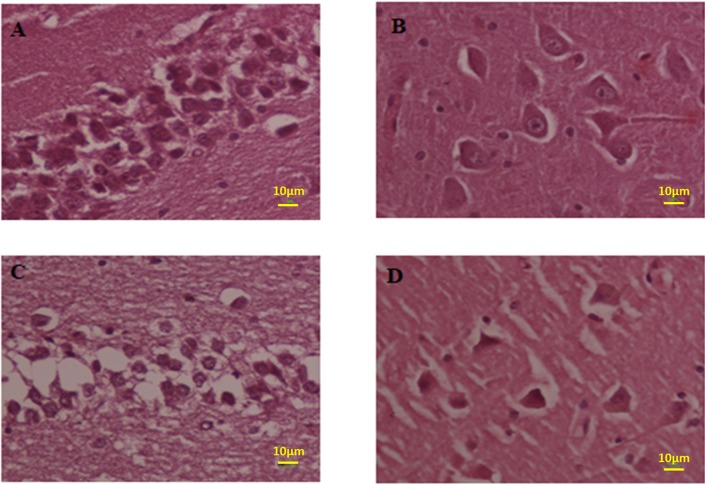
Photomicrograph of the hippocampus stained with H and E. (A and B) Normal and (C and D) Meth groups

**Figure 9:**
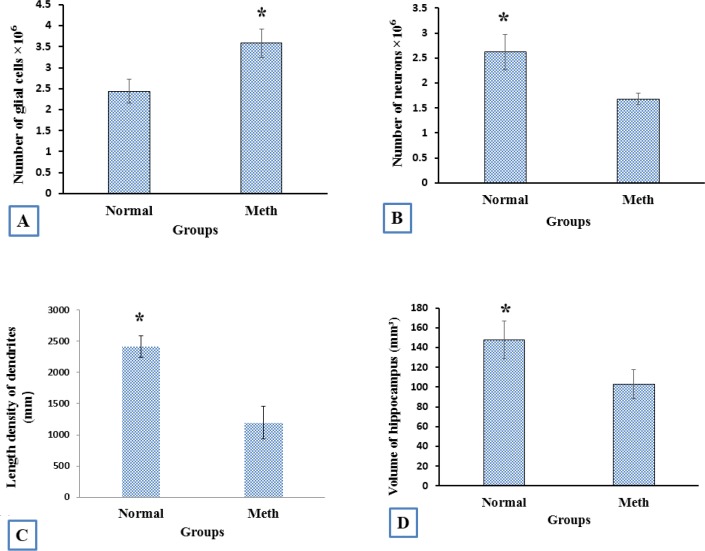
The stereological analysis. (A) the total number of glial cells in Meth groups increased in comparison with the Normal groups (*P* < 0.001). (B) The total number of neuron cells in Meth groups decreased in comparison with the Normal groups (*P* < 0.001). (C) The total length of dendrites in Meth groups decreased in comparison with the Normal groups (*P* < 0.001). (D) The total volume of CA1hippocampus in Meth groups decreased in comparison with the Normal groups (*P* < 0.001)

**Figure 10:**
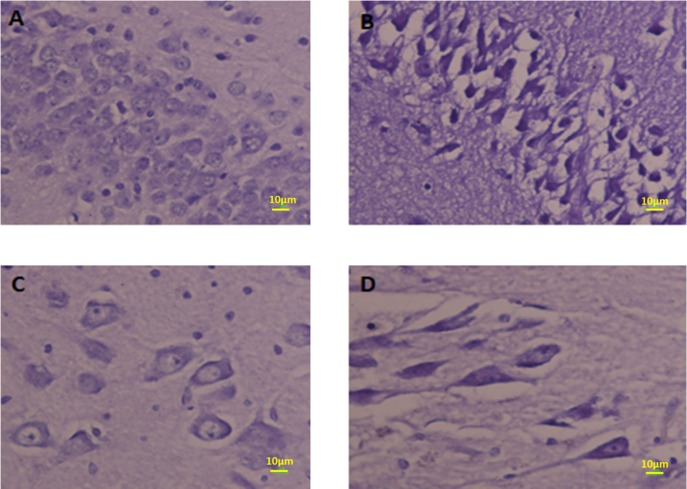
Photomicrograph of the CA1 hippocampus stained by Nissl staining. The neurons in CA1 in Meth groups are shrunk and pyknotic (B and D) but in Normal groups, neurons are normal (A and C).

**Figure 11:**
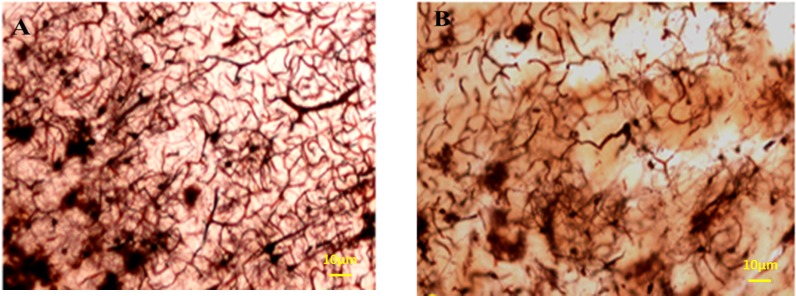
Photomicrographs of the hippocampus stained by Golgi staining. (A) Normal and (B) Meth groups; the total length of dendrites was also decreased in Meth groups in comparison with the Normal groups

The total length of dendrites was also decreased in the Meth groups in comparison with the Normal groups (*P* < 0.001); (Figures 8-11).

*Meth up regulated GFAP and Iba1 and four important nodes in the constructed network*

**Figure 12:**
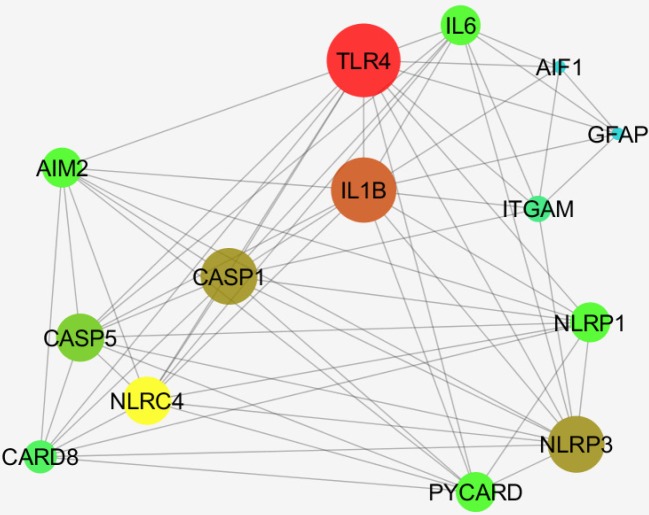
A network including AIF1, Iba1, GFAP and 12 relevant genes are constructed

Among 50 requested additional neighbor genes, only 12 ones were found and added to AIF1 and GFAP (Figure 12). 

Based on the degree value, TLR4, IL1B, CASP1, and NLRP3 are the four important nodes in the constructed network. As it is depicted in Figure 12, ITGAM is the potent mediate between GFAP and AIF1.

## Discussion

Postmortem molecular and histological analyses demonstrated that chronic Meth usage had an adverse effect on the Blood-Brain Barrier (BBB) and CA1 hippocampal region. Astrocytes are the dominant glial cells that outnumber the neurons in the CNS and have been identified as a vital element in the function of blood-brain-barrier (BBB) (28). In several studies, alterations of BBB have been investigated before and after exposure to Meth (29-31). Following the exposure to Meth, the BBB function is modified due to the changes in astrocyte reactivity which ultimately lead to the diminished protection against any further toxicities, oxidative stress through glutathione production (32, 33) ammonia toxicity (34), and also to the regulation of inflammatory responses (35-37). 

With regards to the changes in the CA1 region, one of these adverse effects of Meth is the elevation of GFAP and Iba-1 proteins expression in the CA1 region of Meth users’ hippocampus. The increased GFAP expression in the hippocampus of the chronic Meth users suggests that the hippocampus is susceptible to the disruptions in the blood-brain barrier which results in cytotoxic edema. Previous findings have demonstrated that the gene expression of GFAP remains significantly elevated after 32 days of exposure to a Meth neurotoxic regimen (9). We showed that chronic Meth use induces significant activation of the astrocytes. According to the data obtained from the experimental studies of Meth-induced toxicity, intense microglial and astrocyte activation have been observed (14, 34 and 38). In fact, these changes in the activation of astrocytes and microglia can clearly reflect a neurodegenerative response to Meth. GFAP immunohistochemistry is a widely used method for assessment of astrocyte reactivity and reactive astrocytosis following several CNS pathologies (39, 40). According to another study, the neurotoxic dosage of Meth induces a sharp increase in GFAP in the subgranular zone of the Dentate gyrus which is expressed in the process of neurodegeneration (41). 

As mentioned above, Meth-induced toxicity is also related to the activation of microglia in several different areas of the affected brain (42, 43). Activated microglia have been detected in the brains of animals and humans who were exposed to Meth (44-47). Thomas *et al*. have demonstrated that after treating animals with a neurotoxic Meth regimen, leaving them to recover for 7 days, and then exposing them with another neurotoxic regimen of Meth, they had no other significant microglial activation (48). Microglia is the primary antigen-presenting cells in the CNS and they can migrate to the site of injury after changing their morphology, and start secreting their specific proinflammatory cytokines or some other factors that may trigger and develop inflammation in the brain tissue (49-51). By activation of microglia, several cytokines, ROS and RNS have been expressed subsequently which may be implicated in increasing neurotoxicity. Actually, activated astrocytes and/or microglia may induce releasing of cytokine and disruption in the recovery of function which follows the Meth-induced toxicity. Reactivated microgliosis is a specific marker for severe damage to dopamine terminals after a neurotoxic Meth regimen. However, a critical question remains unanswered concerning the exact role of reactive microgliosis in contributing or mirroring Meth-induced neurotoxicity (9). Based on the previous data, we can conclude that in comparison with astrocytes, the activation of microglia has a greater association with the acute toxic effects of Meth (52). We cannot eliminate the potential character of persistent astrocyte activation in promoting resistance to acute Meth-induced neurotoxicity. On the other hand, it has been proved that astrocytes have a crucial role in the normal functioning of the brain, such as neurotransmission. Changes in their function have been detected in several diseases of the central nervous system. Our histological and stereological results showed that glial and neuronal alternations occur in Meth users’ hippocampus. Our result revealed an increase in the neuron shrinkage and pyknotic changes in the CA1 region and a decrease in the total volume of the hippocampus of the Meth groups. A total number of the neuron and glial cells and the total length of dendrites in Meth groups also decreased in comparison with the Normal groups. These results confirm the toxic effects of Meth on the CA1 hippocampal tissue, which has not been reported for human specimens. Understanding the precise role of neuroglial cells in Meth-induced toxicity is a crucial step in identifying key factors that contribute to or mitigate Meth-induced damage. Glia cells could be targeted for the treatment of Meth addiction based on several studies. For instance, in a postmortem study by Arezomandan *et al*. it has been indicated that glial activation could be implicated in the maintenance and reinstatement of Meth-seeking behaviors. It clarified the role of glial cells in these processes particularly in the maintenance and reinstatement of Meth-induced conditioned place preference (CPP) in rats (53). Inflammation and gliosis have a prominent role in Meth users. The GFAP plays a critical role in astrogliosis in neurodegeneration and on the other hand Iba1 is critical in neuroinflammation. By investigating the four crucial genes in the protein network of GFAP and Iba1, It is evident that they are completely correlated with each other and involved in the immune system and apoptosis as well (54). A new feature of the molecular mechanism of the effects of the Meth in CA1 hippocampus is explored by this investigation. The critical role of ITGAM (highlighted as intramembranous protein) which mediate these processes with the query genes is significant (55).

## Conclusion

Chronic Meth uses probably induce GFAP and Iba-1 upregulation and neuronal apoptosis in the CA1 region of the postmortem hippocampus.
